# Promoting Equity In Clinical Decision Making: Dismantling Race-Based Medicine

**DOI:** 10.1377/hlthaff.2023.00545

**Published:** 2023-10

**Authors:** Tina Hernandez-Boussard, Shazia Mehmood Siddique, Arlene S. Bierman, Maia Hightower, Helen Burstin

**Affiliations:** Stanford University, Stanford, California.; University of Pennsylvania, Philadelphia, Pennsylvania.; Agency for Healthcare Research and Quality, Rockville, Maryland.; University of Chicago, Chicago, Illinois.; Council of Medical Specialty Societies, Washington, D.C.

## Abstract

As the use of artificial intelligence has spread rapidly throughout the US health care system, concerns have been raised about racial and ethnic biases built into the algorithms that often guide clinical decision making. Race-based medicine, which relies on algorithms that use race as a proxy for biological differences, has led to treatment patterns that are inappropriate, unjust, and harmful to minoritized racial and ethnic groups. These patterns have contributed to persistent disparities in health and health care. To reduce these disparities, we recommend a race-aware approach to clinical decision support that considers social and environmental factors such as structural racism and social determinants of health. Recent policy changes in medical specialty societies and innovations in algorithm development represent progress on the path to dismantling race-based medicine. Success will require continued commitment and sustained efforts among stakeholders in the health care, research, and technology sectors. Increasing the diversity of clinical trial populations, broadening the focus of precision medicine, improving education about the complex factors shaping health outcomes, and developing new guidelines and policies to enable culturally responsive care are important next steps.

As the use of artificial intelligence has spread rapidly throughout the US health care system, concerns have been raised about racial and ethnic biases built into the algorithms that often guide clinical decision making.^1^ Algorithms that use race as a proxy for biological differences fail to account for genetic diversity within racial and ethnic groups, and they reinforce stereotypes.^1^ *Race-based medicine* refers to medical practice guided by algorithms that are biased in this manner.^2^ Although data-driven approaches to clinical decision making may be intended to improve the quality and effectiveness of care, they have the opposite effect when they are based on flawed algorithms. Reliance on algorithms that use race as a proxy for biological differences leads to inaccurate estimates of clinical risk and contributes to treatment patterns that are inappropriate, unjust, and harmful to minoritized racial and ethnic groups. These patterns have contributed to persistent racial and ethnic disparities in health and health care in the US.^3^

To advance equity in health care delivery, we recommend a race-aware model for clinical algorithms that is informed by considerations of environmental factors such as structural racism and social determinants of health. In this article we first debunk the flawed assumptions inherent in race-based medicine and illustrate its harmful implications for minoritized racial and ethnic groups. Second, we show how the race-aware approach to clinical decision support can help reduce racial and ethnic disparities in care. We review ongoing efforts to develop more equitable and inclusive approaches to clinical decision making, and last, we recommend next steps toward the goal of replacing race-based medicine with the race-aware approach.

## Flawed Assumptions About Racial And Ethnic Differences

Decisions to use race and ethnicity in algorithms as a marker of biological differences are based on the assumption that people of different racial or ethnic backgrounds have different biological characteristics, predisposing certain racial and ethnic groups to specific health conditions or treatment responses. However, in fact, evidence suggests that genetic differences within the same racial and ethnic group may be greater than those across groups.^4^ Belief that race or ethnicity alone can explain the risk for disease or a response to treatment overlooks the complex interplay of genetic and environmental factors that contribute to disease. This may perpetuate unjust treatment and hinder efforts to provide health care tailored to individuals’ unique needs.

## Disparities Resulting From Race-Based Medicine

Algorithms in which race is a proxy for biological differences often have been used to guide treatment recommendations and resource allocation.^5^ However, such practices can lead to disparities in health care use and outcomes.^5^ For example, race-based medicine has led to inequities in the treatment of kidney disease. Historically, calculations of the estimated glomerular filtration rate—a measure of kidney function used in the algorithm to diagnose and monitor kidney disease—has included adjustments for race. The algorithm assumed that Black patients had higher levels of creatinine (a waste product that is an indicator of glomerular filtration rate) than White patients with the same kidney function. This assumption was based on observational studies that found differences in creatinine levels between racial groups but did not account for other factors, such as diet. Recent studies have shown that using a race-based algorithm for estimated glomerular filtration rate may lead to the underdiagnosis and undertreatment of kidney disease in the Black population.^6,7^ Compared with other racial and ethnic groups, Black people are often diagnosed with kidney disease at later stages.^8^ This delayed diagnosis significantly reduces the likelihood that Black patients with kidney disease will be placed on the national waitlist for a kidney transplant. As a result, Black patients often have missed opportunities for kidney transplantation. To address this inequity and move away from race-based clinical decision making, the National Kidney Foundation and American Society of Nephrology convened a task force that removed race from the estimated glomerular filtration rate calculation in September 2021, replacing it with biological factors, including age, sex, cystatin C, and body weight.^9^

Race-based decision making also has led to disparities in use of hospice care and other end-of-life treatment, particularly among racial and ethnic minority groups.^10^ However, race does not capture or represent the complex interplay of social and environmental factors that influence end-of-life needs,^11^ nor does it capture individual patients’ preferences. Moreover, it reinforces stereotypes by assuming that certain racial and ethnic minority groups might not benefit from or desire additional care.

## A Race-Aware Approach To Clinical Decision Support

The inclusion of race and ethnicity in algorithms is problematic when it perpetuates the false notion that race is a biological construct. However, inclusion of race and ethnicity in algorithms to intentionally tackle inequities by accounting for environmental factors such as structural racism and social determinants of health can be beneficial. This race-aware approach acknowledges the role of structural racism in patient outcomes. For example, the kidney allocation system is an algorithm-based protocol used to prioritize patients for kidney transplants on the basis of the amount of time they have been on the national transplant waitlist.^12^ In 2014 the system was re-calculated to consider either the date on which patients were added to the waitlist or the date of first regular dialysis, whichever came first.^12^ This change was intended to benefit racial and ethnic minority populations, who often spent more time on dialysis than White patients before being placed on the waitlist.^13^ After implementation of the revamped kidney allocation system, the overall waitlist rate decreased for all racial and ethnic groups, with a reduction in the difference between Black and White end-stage renal disease waitlisting rates.^12^

In another example of transitioning from race-based medicine to a race-aware approach, in 2021 the Maternal Fetal Medicine Units Network removed race as a risk factor for poor outcomes in the vaginal birth after cesarean calculator.^14^ This algorithm is used for clinical decision support to identify women at low risk from complications if they choose vaginal birth. When race was used as a risk factor in the calculator, women who self-identified as Black or Hispanic had half the odds of being recommended for vaginal birth after cesarean. However, in 2021, evidence demonstrated that differences in risk among Black and Hispanic women were explained by their higher prevalence of chronic hypertension.^2^ The revised algorithm, in which history of chronic hypertension replaces race as a risk factor,^15^ offers a more accurate assessment of the risk associated with vaginal birth after cesarean for all patients, while removing the race-based disparity. This change demonstrates the benefit of identifying factors beyond race and ethnicity that explain variations in health outcomes. Ongoing clinician and patient education is needed to achieve widespread use of the revised algorithm.

## Toward Unbiased Algorithms

Algorithms can exacerbate or perpetuate health disparities even when they do not use race explicitly as a risk factor or a proxy for biological differences. For example, one widely cited study published in 2019 found that Black patients were less likely than their White counterparts to be enrolled in a care management program, even when Black patients were sicker.^3^ This disparity was attributed to an algorithm that used prior health care use as a proxy for health care need—but the lower prior use seen among Black patients was largely a consequence of the barriers they faced in accessing health care services. To identify and eliminate algorithmic bias, it is important to carefully assess all variables in the underlying assumptions and determine how each contributes in the causal pathway to outcomes.

## Efforts To Develop More Equitable And Inclusive Approaches To Clinical Decision Making

### POLICY CHANGES IN MEDICAL SPECIALTY SOCIETIES

Medical specialty societies across the country increasingly have recognized the harms caused by race-based medicine and have taken action in the past several years to end the consideration of race in clinical algorithms. For example, to address the inequity in diagnosis of pulmonary disease, whereby Black patients were disproportionately undiagnosed and underdiagnosed, a workshop panel appointed by the American Thoracic Society determined in 2023 that race and ethnicity should not be considered in the interpretation of pulmonary function tests.^16^ This policy is known as a “race-neutral approach.” Striking the right balance between the race-neutral approach, which avoids using race as a risk factor in clinical decision making, and the race-aware approach, which incorporates data on disparities in an effort to advance health equity, is essential to improving outcomes for all patients, regardless of their racial or ethnic background.When used to advance equity, algorithms should consider all factors affecting access and utilization, including race and ethnicity. The responsible use of algorithms includes taking precautions to avoid perpetuating bias or reinforcing stereotypes.

In a broad-based approach to dismantling race-based medicine, the American Academy of Pediatrics made the decision in 2020 to eliminate race as in input in all of its clinical algorithms and guidelines used in clinical practice.^2^ Black race had previously been considered a protective factor against urinary tract infections in children, despite a large body of evidence to the contrary.^17^ This erroneous assumption led to delayed or inadequate treatment of urinary tract infections among Black children compared with their counterparts from other racial and ethnic backgrounds. The American Academy of Pediatrics’ decision to eliminate race-based medicine was coupled with its stated “commitment to dismantle the structural and systemic inequities that lead to racial health disparities.”^2(p1)^

### CHANGING METHODS OF ALGORITHM DEVELOPMENT

As the limitations and harms of race-based medicine have become increasingly clear, stake-holders in the health care, research, and technology sectors have sought more equitable and inclusive approaches to clinical decision making. To reduce algorithmic bias, during the past few years data scientists have sought to engage diverse teams in the process of algorithm development.^18^ Training algorithms on data that reflect the population’s racial and ethnic diversity also can reduce the risk for bias.^19^ Use of publicly available source code enables stakeholders in health care, research, and technology to scrutinize, identify, and address any potential biases or disparities in outcomes associated with clinical algorithms.^20^ To correct algorithmic biases in automated clinical decision processes, data scientists have increasingly sought mathematical solutions. These approaches include evaluations of model performance to determine how positive outcomes are distributed across subpopulations.^21^ Adjustments are made as needed to promote equity. Use of the equalized odds ratio, which balances true-positive and false-positive rates of algorithmic outcomes across demographic groups, can help achieve comparable algorithmic performance rates for each demographic group, thus ensuring that algorithms perform consistently and equitably.^21^ Although these sociotechnical approaches to mitigating algorithmic bias have been adopted at some leading academic institutions, widespread adoption remains sporadic and inconsistent across the broader community. Because racial and ethnic biases can be introduced at all stages of an algorithm’s life cycle, broad-based reform efforts are needed. In the following section, we describe important next steps to advance equity in clinical decision making.

## Dismantling Race-Based Medicine: A Road Map For Reform

Race-based medicine is a complex and deeply entrenched problem in the US health care system. Success in dismantling race-based medicine will require continued commitment and sustained efforts among stakeholders in health care, research, and technology. Next steps should focus on four areas.

### INCREASE THE DIVERSITY OF CLINICAL TRIAL POPULATIONS

Evidence from clinical trials informs clinical guidelines, which are incorporated in algorithms for decision support. The historical underrepresentation of racial and ethnic minorities in clinical trials has resulted in guidelines and algorithms that might not accurately represent the entire population.^22^ To dismantle race-based medicine, it is crucial to have trial data that include populations representing diverse racial and ethnic groups. Trial diversity allows for the identification and understanding of outcomes and risk factors beyond race and ethnicity, including social determinants of health. By considering these factors in algorithm development alongside race and ethnicity, researchers gain valuable insights into the factors’ complex relationships with disease progression and treatment response. Incorporating data related to social determinants of health in algorithms also can enable effective strategies for disease management, treatment adherence, and improved health outcomes across diverse populations. Active involvement of racial and ethnic minority communities in the design and implementation of clinical trials can help overcome historical mistrust and hesitancy to participate in medical research.

### BROADEN THE FOCUS OF PRECISION MEDICINE

Precision medicine tailors medical treatment to individual patients’ characteristics and has the potential to improve health outcomes. However, because precision medicine often is narrowly focused on individuals’ genetic characteristicsand does not address other critical factors thatinfluence health outcomes, it may help perpetuateracial and ethnic health inequities.^23^ In contrast to precision medicine, precision care is an approach that considers patients’ multiple morbidities functional status, values, goals, and preferences, as well as their social and societal contexts, in the process of developing treatment recommendations.^23^ By accounting for social and environmental factors such as income and educational attainment, precision care can help reduce disparities and advance equity.We recommend broadening the focus of precision medicine to align with the precision care approach.

### PROMOTE EDUCATION AND AWARENESS

To move past oversimplified and inaccurate assumptions about race and health, it will be important to increase education among health care professionals, researchers, and the public about the complex social, economic, and environmental factors that shape health outcomes. Education efforts should aim to deepen understanding of the ways in which racism and discrimination contribute to health disparities, and they should emphasize the need to address structural inequalities in health care.

A growing body of research suggests that education and awareness-raising efforts can be effective in challenging race-based medicine and promoting health equity. For example, a 2020 study found that an educational intervention for medical students that emphasized the effect of social determinants of health on outcomes led to increased awareness of health disparities.^24^

### DEVELOP GUIDELINES AND POLICIES TO ENABLE CULTURALLY RESPONSIVE CARE

To move past a race-based approach to medicine, there is a need for public- and private-sector guidelines and policies to increase the collection of comprehensive data on environmental factors, such as social determinants of health, that affect health outcomes. Policies and guidelines should focus on standardized data collection protocols and integration of data on social determinants in electronic health records during routine patient encounters. Increased availability of patient-specific data on social determinants at the point of care will help health care providers recognize and address the unique needs and challenges faced by diverse patient populations.

## Conclusion

The United States has a long history of using race in medical decision making, thus contributing to health disparities and perpetuating biases. Progress is being made to dismantle race-based medicine, but more work is needed to ensure that health care is delivered in a fair and equitable manner for all. As these efforts move forward, widespread adoption of a race-aware approach to care holds the promise of fostering a more inclusive and equitable health care system in which every person receives personalized and compassionate care that respects their diverse backgrounds, values, and needs.
